# Xylem, phloem and transpiration flows in developing European plums

**DOI:** 10.1371/journal.pone.0252085

**Published:** 2021-05-20

**Authors:** Andreas Winkler, Moritz Knoche

**Affiliations:** Institute for Horticultural Production Systems, Leibniz-University Hannover, Hannover, Germany; Universidade do Minho, PORTUGAL

## Abstract

Neck shrivel is a quality disorder of European plum (*Prunus × domestica* L.). It has been suggested that backflow in the xylem (from fruit to tree) could contribute to the incidence of neck shrivel in plum. The objective was to quantify rates of xylem, phloem and of transpiration flow in developing plum fruit. Using linear variable displacement transducers, changes in fruit volume were recorded 1) in un-treated control fruit, 2) in fruit that had their pedicels steam-girdled (phloem interrupted, xylem still functional) and 3) in detached fruit, left in the canopy (xylem and phloem interrupted). Xylem flow rates were occasionally negative in the early hours after sunrise, indicating xylem sap backflow from fruit to tree. Later in the day, xylem flows were positive and generally higher in daytime and lower at night. Significant phloem flow occurred in daytime, but ceased after sunset. During stage II (but not during stage III), the rates of xylem flow and transpiration were variable and closely related to atmospheric vapor pressure deficit. The relative contribution of xylem inflow to total sap inflow averaged 79% during stage II, decreasing to 25% during stage III. In contrast, phloem sap inflow averaged 21% of total sap inflow during stage II, increasing to 75% in stage III. Our results indicate that xylem backflow occurs early in the day. However, xylem backflow rates are considered too low to significantly contribute to the incidence of neck shrivel.

## Introduction

Neck shrivel is a physiological disorder of European plum that occurs towards maturity and continues to develop in postharvest. A loss of turgidity results in shriveling of the pedicel end of the fruit, while the stylar end remains turgid. These symptoms reduce the visual quality of the fruit and thus result in lower prices at market. Neck shrivel has a genetic component. Susceptible cultivars include ‘Hauszwetsche’ and its clones and ‘Toptaste’ [1]. However, neck shrivel must also have an environmental component, since the incidence of symptomatic fruit in the susceptible cultivars varies from season to season. Anecdotal evidence suggests high temperatures and low relative humidities (RH) are conducive to neck shrivel [2]. European plum is not the only fruitcrop species where preharvest shrivel symptoms have been reported. Shrivel has also been reported in sweet cherry [3], kiwifruit [4] and grapes [5,6].

Increased transpiration through microcracks in the pedicel region has been identified as a factor in neck shrivel in plum [1]. The microcracks were radially oriented and mainly limited to the pedicel end of the fruit. The reasons for the distinct microcrack orientation and for their asymmetrical location in a fruit of roughly symmetrical shape are not known. Also, it is not known, whether the microcracking at the pedicel end of the fruit is the only factor in neck shrivel or whether other factors are also involved.

An additional explanation for neck shrivel could be a negative xylem flow (‘backflow’) from the fruit to the tree. The occurrence of xylem backflow has been demonstrated in apple [7], grape [8], kiwifruit [9] and—to some extent—also in sweet cherry [10]. If xylem backflow also occurs in plum, and if it also occurs during late fruit development, then it too may contribute to the dehydration of the pedicel end of the fruit and, hence, to neck shrivel [1]. To our knowledge, there is no published information on vascular flows in developing (European) plum. For Japanese plums, vascular flows at maturity were reported recently [11].

Vascular flows can be analyzed *in situ* using linear variable displacement transducers (LVDTs) [10]. The LVDTs record changes in fruit diameter. Using an appropriate geometrical model for fruit shape, a measured change in fruit diameter (mm) may be used to calculate a corresponding change in fruit volume (V, mm^3^). For a fruit with few airspaces (most of the volume of a plum is water), any change in fruit volume over a defined period of time measures the algebraic sum of all the water flows into it and out of it over that period. These flows occur in the xylem sap, in the phloem sap and through the skin by transpiration (in the dry) and by osmosis (in the wet). Next, by manipulating the sap flows in the fruit pedicel using steam girdling or detached fruit as proposed by Lang and Thorpe [12] and Lang [7], a change in V per unit time can be partitioned into three flow components: xylem flow (X), phloem flow (P) and transpiration flow (T). The method is based on the assumptions that (1) the rate of transpiration is not affected by detachment of the fruit and (2) the xylem flow is not affected by the steam girdling. While the first condition is generally fulfilled (e.g. [10]), the second condition does not always hold. According to [13], steam girdling decreases conductance and this may lead to errors in the calculated flows during periods when fruit growth is limited. These are the early morning or early evening hours, whereas during most of the day (decreasing fruit volume) or night (increasing fruit volume), the resulting error is small [13]. Also, to date, this is the only method which allows to estimate vascular and transpiration flows in the field on a statistically sound number of fruit [14,15].

The objectives of this study were 1) to quantify the diurnal patterns of flows in xylem, phloem and transpiration during the development of a European plum and 2) to examine the possibility that a putative xylem ‘backflow’ could contribute to neck shrivel.

## Materials and methods

### Plant materials

Experiments were carried out *in situ* in field-grown plum trees (*Prunus x domestica* L.) of the cultivar ‘Hauszwetsche Wolff’ at the Horticultural Research Station of the Leibniz University in Ruthe, Germany (lat. 52°14’N, long. 9°49’E). Trees were grafted on ‘St Julien A’ rootstocks. Standard cultural practices for irrigation, fertilization and pruning were applied by our experienced staff. Measurements were made between 55 and 147 days after full bloom (DAFB). Fruit selected for measurement were of uniform developmental stage, as indexed by size and color, and were without visible defects.

### Mass, color and osmotic potential

Fruit mass, color and the osmotic potential of the expressed juice were determined individually on 15–20 fruit throughout the season. Fruit mass was determined gravimetrically, fruit color using a spectrophotometer (CM-2600d; Konica Minolta, Tokyo, Japan) and juice osmotic potential using a water vapor pressure osmometer (VAPRO 5600; Wescor, Logan, UT). Before measuring color, the epicuticular waxes were removed by wiping with soft tissue paper.

### Fruit volume

The fruit volume (V; mm^3^) was estimated based on the average of two orthogonal measurements of equatorial fruit diameter (d; mm) and one of fruit height (h; mm) using a digital caliper. Fruit shape was assumed a spheroid with the following formula:

$$V=\frac{4}{3}*\pi *{\left(\frac{d}{2}\right)}^{2}*\frac{h}{2}.$$

It was further assumed, that the measured (LVDT) change in d was accompanied by a proportional change in h, such that the ratio d to h remained constant during any 6-d measurement period (i.e. that fruit shape was conserved over this period). It was also assumed, that the density of the fruit is 1, such that the change of volume was given in μl or ml (1 mm^3^ = 1 μl).

### Xylem, phloem and transpiration flows

Flows were determined as described previously [10]. The plum was positioned in a Lucite frame such that one side was in contact with the frame and the opposite side with an LVDT (SM277.15.2.STX24, resolution 5 μm; Schreiber Meßtechnik, Oberhaching, Germany). In this arrangement, the LVDT quantified changes in fruit equatorial diameter. Temperature and relative humidity (RH) were monitored within the canopy (CS215; Campbell Scientific, Shepshed, UK). Based on these data, the vapor pressure deficit (VPD) was calculated [16]. Data were logged at 5-min intervals (CR3000 data-logger, Campbell Scientific).

First, a developmental time course was established using 27 LVDTs mounted as above. Data were recorded for 72 h. After this time, each LVDT and its fruit was assigned to one or other of three groups of 6 to 9 fruit each (27 fruit in all). Fruit of the first group (C) served as un-treated controls. For a control fruit, the volume change equals the sum of xylem (X) in- and outflows, the phloem flow (P) and the transpiration (T). Note that T takes a negative sign (i.e. T flow is out of the fruit), also T can sometimes include a small positive component of osmotic water uptake through the skin into the fruit. The pedicels of fruit of the second group (G) were steam-girdled using a steam generator (SC952; Alfred Kärcher, Winnenden, Germany). Care was taken not to allow steam to damage the fruit itself. With steam-girdling, all cells in the steamed region of the pedicel are killed and phloem transport (a living process) is interrupted, whereas xylem transport (a non-living process) is unaffected [7,17]. Fruit from the third group (D) were detached by cutting the pedicel thereby interrupting X and P. The cut end of the pedicel was sealed using a fast-curing epoxy glue (UHU plus schnellfest; UHU, Bühl/Baden, Germany). The fruit remained in the same position in the canopy, such that the transpiration of the detached fruit (D) was considered representative of that of the still-attached fruits (C and G). It is worth noting that the skins of stage III stone fruit contain only very few and mostly inactive stomata [18], so their transpiration is unlikely to be affected significantly by treatments applied to their pedicels over the time periods of these manipulations. From the above discussion, it follows that the value of X is the difference in volume change between a steam-girdled fruit (G) and a detached fruit (D) (Eq 1), and also that the value of P is the difference in volume change between an un-treated control fruit (C) and a steam-girdled fruit (G) (Eq 2), whereas the detached fruit (D) represents the transpiration T (Eq 3).

X=G−D (1)

P=C−G (2)

T=D (3)

Transpiration of the detached fruit decreased their diameters, so transpiration flows usually had a negative sign. Occasionally, transpiration was found to have a positive sign. This indicates surface water uptake via the skin must have occurred during dew/rainfall. The diameter changes of the fruit of the three groups (C, G, D) were recorded for a further 72 h period and the corresponding changes in fruit volume were calculated using treatment means and the above equation. An estimate of the standard error (SE) of the differences *G–D* (*SE*_*X*_) was then obtained using the following equation (Eq 4). In this equation, the *SE*_*G*_ and *SE*_*D*_ represent the *SEs* for the means of *G* and *D* [19]:

$${SE}_{X}=\sqrt{{SE}_{G}^{2}+{SE}_{D}^{2}}$$ (4)

The *SE*_*P*_ was calculated accordingly using the *SE*_*C*_ and *SE*_*G*_. Occasionally, a replicate was lost due to slippage in the Lucite frame caused by rain or wind or by insect damage. An exception was in late stage III of fruit development, where only four fruit remained in the D group. Transpiration flows were further analyzed by calculating the flux density by dividing the transpiration flow through the skin, by the fruit surface area. To normalize for differences in the transpiration driving force, flux density was then divided by the VPD (estimated from the temperature and RH values measured in the canopy). The normalized flux density is referred to as the skin permeance.

### Data analysis

The 72 h period after treatment by steam girdling or by detachment was divided into three 24 h intervals and the X, P and T flows calculated for anyone of the three intervals. Calculations were based on the means of the four to eight fruits in the groups C, G and T. These data were used to calculate the correlations and time courses depicted in Figs 4–6. For Fig 7, the means of the three 24 h intervals were averaged. For data on single replicates the reader is referred to S1 Fig.

**Fig 1:**
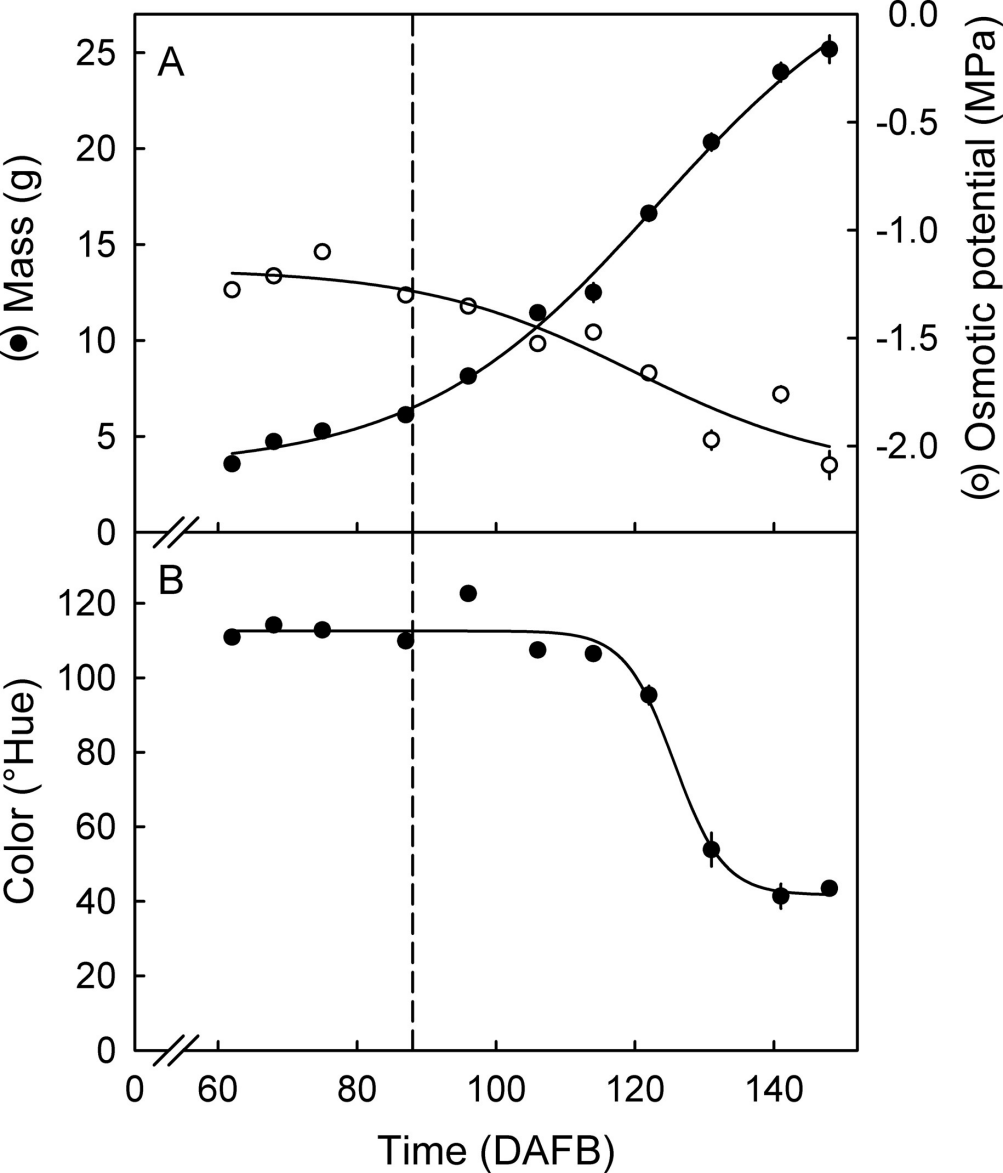
Fruit mass, expressed fruit juice osmotic potential (A) and fruit skin color (B) of developing European plum. Fruit skin color is indexed as a change in Hue angle. The vertical dashed line indicates the transition between stage II and stage III. Time (*x*-axis) is in days after full bloom (DAFB). Data represent means ± SE.

Data in Figs 1, 4, 5, 6A and 7 are presented as means ± SE. When no error bar is visible, the SE is smaller than the plotting symbol.

Data were analyzed by correlation and regression analyses (R 3.6.1; R Foundation for Statistical Computing, Vienna, Austria). Significances of the coefficients of determination (r^2^) at the 0.05 and 0.01 probability levels are indicated by * and **, respectively.

## Results

During development, fruit mass increased sigmoidally with time, while osmotic potential decreased (Fig 1A). Over the same period, fruit color changed from green (~110°Hue) to reddish/purple (~40°Hue), the color change being relatively abrupt at about 120 DAFB (Fig 1B).

**Fig 2:**
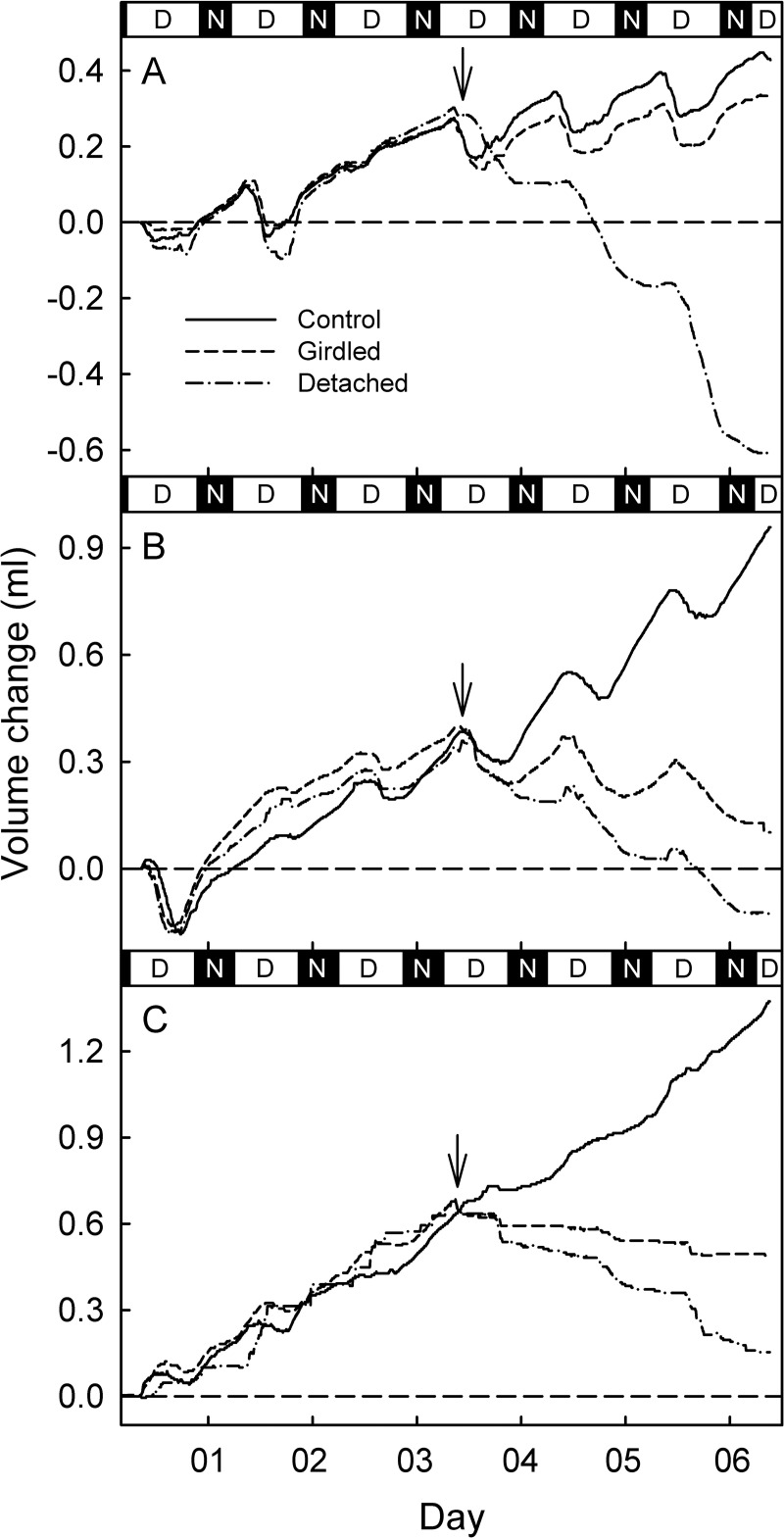
Typical weekly time courses of change in fruit volume calculated from changes in the three orthogonal diameters of European plum fruit during stage II at 75 days after full bloom (DAFB) (A), in early stage III (103 DAFB) (B) and in late stage III (132 DAFB) (C). In the morning of the fourth day (indicated by arrow), fruit pedicels were steam-girdled (dashed line) or detached (dashed and dotted line), but all fruit remained *in situ* in the canopy. Un-treated fruit served as controls for comparison. Data are for a single fruit representative for the steam-girdled, detached and control treatments. D and N on the *x*-axis indicate day (light) and night (dark) periods.

During stage II (Fig 2A) and early stage III (Fig 2B) fruit volume followed a sinusoidal pattern aligned with the day/night cycle. The fruit usually shrinking during the day and expanding at night with the nocturnal expansion being generally greater than the diurnal shrinkage, so the net result over any 24-hour period was a small increment of growth. The largest daily volume usually occurred in the early morning. This sinusoidal pattern almost disappeared during late stage III (Fig 2C). During stage II, the steam-girdled fruit showed a pattern of volume change similar to the un-treated control fruit, but at somewhat reduced level (Fig 2A). During early and late stage III, the volumes of steam-girdled fruit showed a net decrease (Fig 2B and 2C). The volume of the detached fruits decreased continuously. The decreases were larger during the day and smaller during the night (Fig 2).

**Fig 3:**
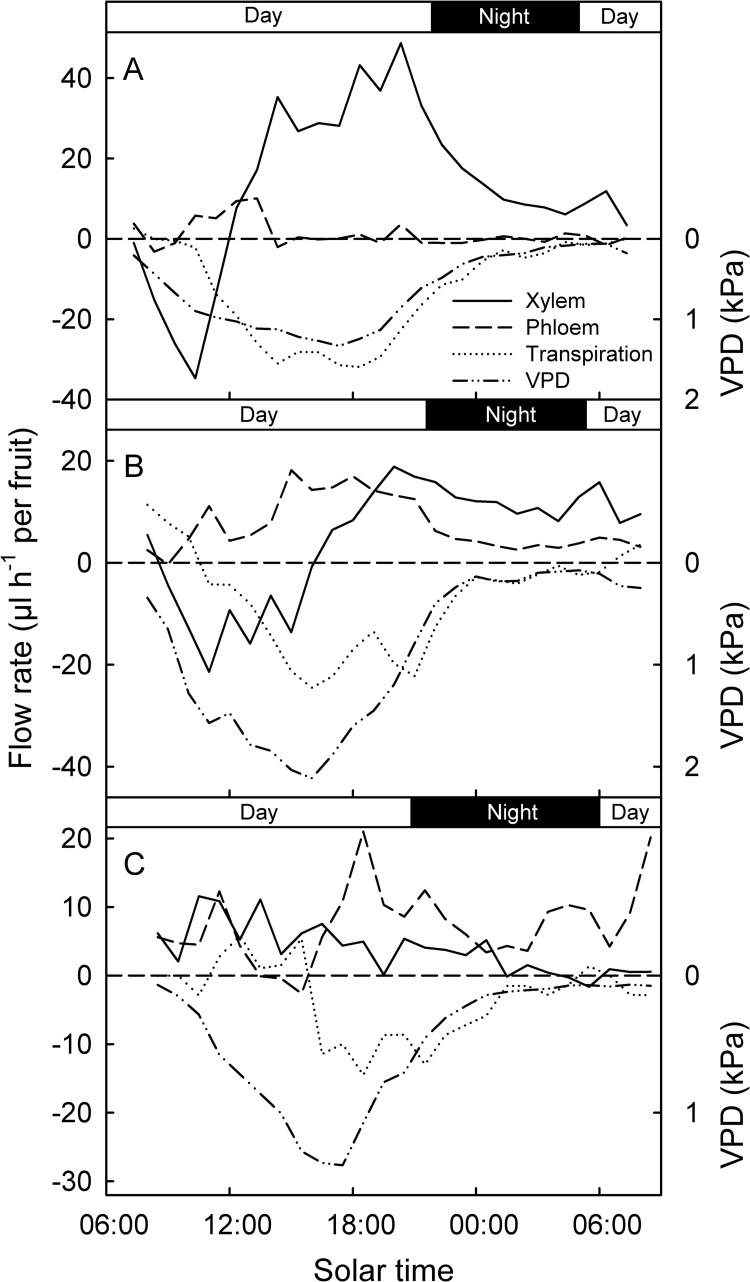
Representative diurnal time courses of xylem, phloem and transpiration flow rates in developing European plum fruit (A: Stage II, 76 days after full bloom (DAFB), B: Early stage III, 104 DAFB, C: Late stage III, 132 DAFB). The pattern of change in water vapor pressure deficit (VPD) is given for comparison. Xylem, phloem and transpiration flows were calculated from net flows determined for un-treated control fruit, steam-girdled fruit and detached fruit. For details see Materials and Methods.

The X, P and T flows followed a characteristic pattern during the course of a typical day (Fig 3). In stage II and in early stage III, and occasionally also in late stage III (S2 Fig), X flows were negative during several hours until the early afternoon, indicating a xylem ‘backflow’ from fruit to tree. Later in the day, X flows became positive (from tree to fruit) and remained high until sunset. At night, X flow rates decreased rapidly until sunrise next morning. The daily X flow rates were higher during stage II than during early and late stage III. The P flow rates were low during stage II and increased during early and late stage III. Significant P flows occurred during daytime, but ceased soon after sunset. In late stage III, peak P flows occurred after sunset, during the early night. At this time, the P inflow rates exceeded X inflow rates.

The diurnal change in T mirrored the diurnal change in VPD, but with a short delay during stage II and a longer delay during late stage III (Fig 3).

**Fig 4:**
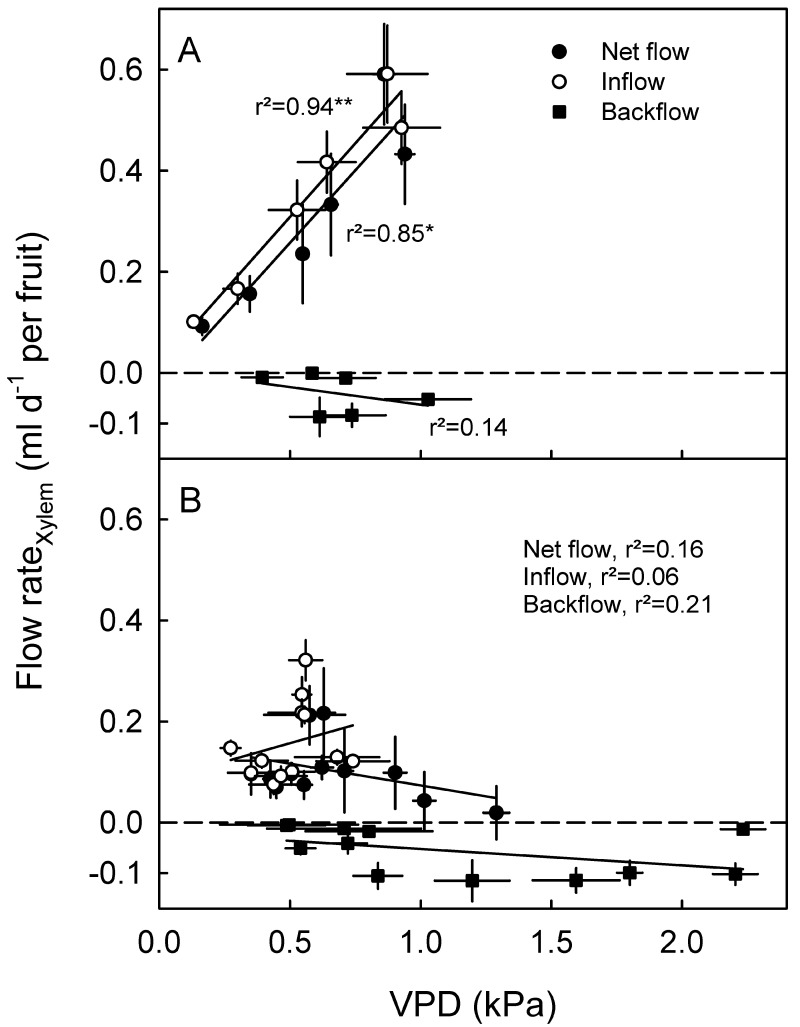
Daily total xylem inflow (positive), total xylem backflow (negative) and net daily xylem flow in stage II (A) and stage III (B) for European plum fruit as affected by atmospheric vapor pressure deficit (VPD). The net xylem flow was calculated as the algebraic sum of the total daily inflows plus the total daily backflows. Because fruit softening may slightly affect the measurement of fruit diameter change by a lightly spring-loaded linear variable displacement transducer, data for the last sampling period were not shown, as this is when fruit softening would have been most pronounced. Data represent means ± SE.

In stage II, there was a significant linear relationship between net flow and inflow to the fruit via the xylem and atmospheric VPD, but not between the xylem backflow and atmospheric VPD (Fig 4A). In stage III, there were no correlations between net flow, xylem inflow or backflow and atmospheric VPD (Fig 4B).

**Fig 5:**
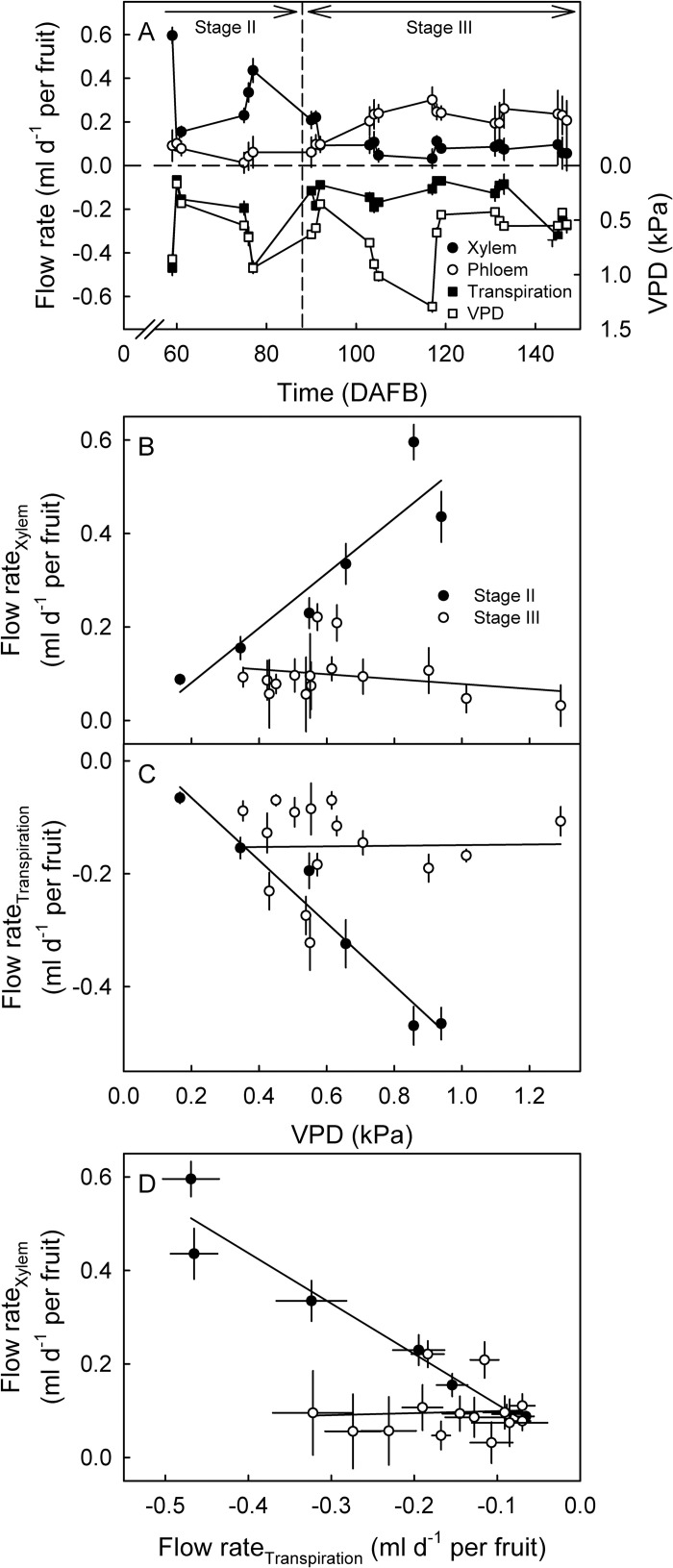
(A) Developmental time course of the water flow rates through the skin (transpiration) and through the pedicel (xylem and phloem) of fruit of European plum. Values of atmospheric water vapor pressure deficit (VPD) are provided for comparison. (B) Xylem flow rates and (C) transpiration flow rates as affected by VPD. (D) Relationship between xylem flow rate and transpiration flow rate. The vertical dashed line in A indicates the stage II/III transition. Xylem, phloem and transpiration flows were calculated from flows determined for un-treated control fruit, steam-girdled fruit and detached fruit. Data represent means ± SE for 24 h time intervals. For details see Materials and Methods.

The developmental time courses of the X and T flows were rather variable, but that of P was less variable. The P flow was about constant during stage II, but increased during stage III up to 118 DAFB and then remained constant (Fig 5A). On average, the X flow rates were higher during stage II, then decreased and remained low throughout stage III (~90 DAFB). The variabilities in X flow and T flow during stage II were largely accounted for by variations in atmospheric VPD (Fig 5B and 5C). However, during stage III, the X and T flows were largely independent of VPD. As expected, the X and T flows were closely interrelated during stage II, but not significantly interrelated during stage III (Fig 5D).

**Fig 6:**
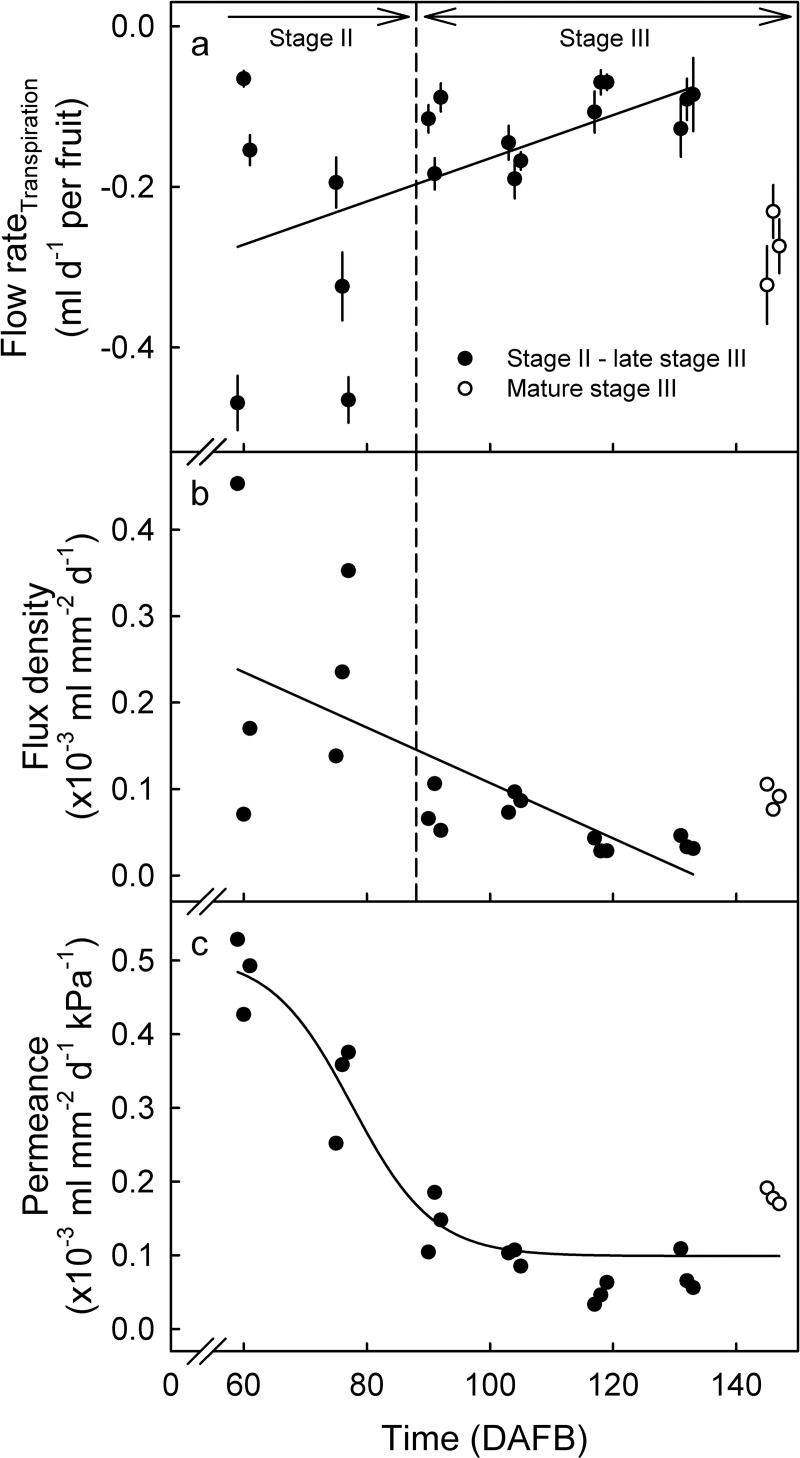
Transpiration of developing European plum fruit. (A) Transpiration rate (per fruit). (B) Transpiration flux density (volume per mm^2^ of skin and day). (C) Skin permeance (flux density per unit water vapor pressure deficit (volume per mm^2^ of skin and day and VPD)). Transpiration flux density was calculated by dividing the flow rate in transpiration per fruit by fruit surface area. To avoid confusion, the absolute of value of the flux density and skin permeance is shown. Skin permeance was calculated by dividing transpiration flux density by the VPD. The open symbols represent the final sampling date. Because fruit softening may slightly affect the measurement of fruit diameter change by a lightly spring-loaded linear variable displacement transducer, data for the last sampling period were excluded from the regression analysis as this is when fruit softening would have been most pronounced. Data in Fig 6A represent means ± SE.

Calculated transpiration flux density showed a marked decrease during development. Variability in transpiration flux density decreased from stage II to stage III (Fig 6A and 6B). Variability in transpiration flux density was further decreased when normalized for changing atmospheric VPD (Fig 6C). The sigmoidal relationship thus obtained shows fruit skin permeance (i.e. skin transpiration flux density normalized for atmospheric VPD is skin permeance) decreases markedly during fruit development.

**Fig 7:**
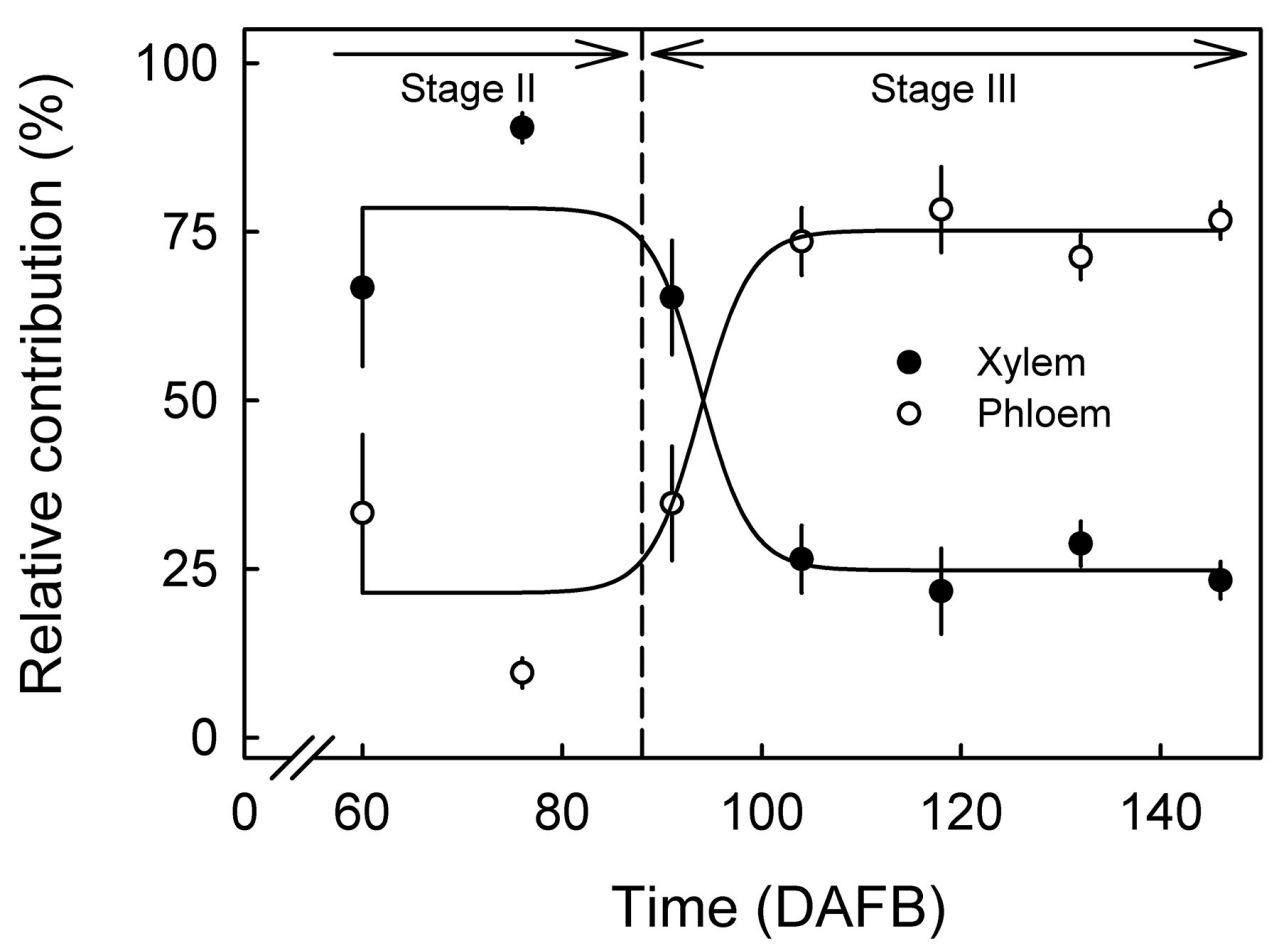
Relative contributions of xylem and phloem sap inflows to total sap inflow to fruit of developing European plums. Total inflow (100%) was calculated as the sum of the xylem and phloem inflows. Data represent means ± SE.

In stage II, the relative contribution of X flow to total vascular flow (X + P) into a fruit averaged 79%. In early stage III the X contribution decreased to 25% and thereafter remained about constant (Fig 7). In contrast, during stage II the P inflow accounted for 21% of total vascular inflow. In early stage III the P contribution increased to 75% and thereafter remained about constant through to maturity (Fig 7).

## Discussion

In our discussion we focus on (1) the xylem, phloem and transpiration flows in the course of development and (2) the xylem backflow and its potential role in neck shrivel.

### Xylem, phloem and transpiration flows in developing fruit

The X flow in European plum decreased during stage III. This conclusion is based on the following observations: 1) The relative contributions of X flow to total flow decreased markedly during development. 2) There was a close relationship between atmospheric VPD (the driving force for transpiration) and X flow during stage II (xylem functional), but not during stage III (xylem largely dysfunctional). 3) The diurnal oscillations of X flow decreased as development proceeded. Oscillations in X flow were essentially absent during late stage III. Our observations are consistent with a loss of functionality of the fruit xylem during the development of European plum. This is in line with similar results for other fleshy fruitcrop species, e.g. apple [20,21], grape [22–29], mango [30], kiwifruit [31,32], sweet cherry [10,33,34] and tomato [35,36]. Only in peach does the xylem seem to remain functional into maturity [37]. Two factors have been identified as contributing to decreases in xylem functionality: 1) a physical disruption of the xylem due to growth strain [21,33,38,39] and/or 2) a blockage of the xylem in the pedicel [29].

Phloem flows were low during stage II, but higher during stage III. This change in flow is accounted for by a high demand for carbohydrates during stage III, when fruit mass increases rapidly [40,41]. During stage II, the pit develops with little change in fruit mass [42,43]. The demand for carbohydrates is low [40,41].

The decrease in fruit transpiration during development was somewhat unexpected. Based on the obvious increase in fruit volume and fruit surface area during stage III, along with a decrease in cuticle mass per unit area and an increase in microcracking [44], we would have expected an increase in fruit transpiration, particularly when normalized by dividing by the increasing fruit surface area (to give a transpiration flux density) and then by dividing by the vagaries of atmospheric VPD (to give a skin permeance). This decrease in skin permeance is not unique for European plum, but was also reported in Japanese plums [11]. These findings are contrary to that for sweet cherry fruit, where skin permeance increases towards maturity, passes through a peak and then decreases [10]. Furthermore, transpiration in plum is closely related to the atmospheric VPD during stage II, but it is almost independent of VPD during stage III. The reason for this is that fruit apoplast water vapor pressure is not well coupled to atmospheric VPD. A decrease in fruit transpiration during development can be accounted for by a decrease in permeance of the fruit cuticle. Similar changes have been reported for developing apricots [45], grape berries [46,47], kiwifruit [48] and nectarines [49]. A decrease in fruit skin permeance must over-compensate any increases in cuticular microcracking that may have occurred. Earlier studies established that wax mass increased markedly during development in European plum [44] and that it is the wax within the cuticle that is responsible for its barrier function [50], whereas accumulation of epicuticular wax does not generally offer a significant barrier to the transpiration of fruit [51] or of leaves [52]. This conclusion also holds for European plum, where mechanical removal of the epicuticular wax bloom had essentially no effect on transpiration rate [53].

### Xylem backflow and its potential role in neck shrivel

During stage II and occasionally during late stage III, we observed negative X flow i.e. a ‘backflow’ from fruit to tree. This backflow usually occurred in the morning hours, just after sunrise. The prerequisites for a X backflow are: 1) at least some xylem functionality must remain and 2) the driving force (a pressure gradient in the xylem) must be in the ‘reverse’ direction (i.e. so as to pull water from the fruit to the tree). Our results reveal that during stage III about 25% of the total water inflow to a plum is in the xylem. This indicates some persistent xylem functionality. The second factor, a ‘reverse’ pressure gradient, most likely results from high foliar transpiration following sunrise that lowers tree water status to the point at which the apoplastic pressure in the tree falls below the value established in the fruit apoplast during the previous hours of darkness. To help explain this, note first, that leaf transpiration (many open stomata) greatly exceeds fruit transpiration (few, non-functional stomata for example see [18]). Second, that the hydraulic capacitance of a leaf is low (small volume and a relatively rigid structure), whereas the hydraulic capacitance of a ‘soft’ fruit is much higher (a very much larger volume and a relatively flexible structure). Third, fruit are often surrounded and occluded by leaves. This will generate a higher-humidity and a lower-light environment around the fruit, so decreasing the driving force for fruit transpiration relative to leaf transpiration. Under these circumstances a backflow from fruit to tree is likely to occur. This scenario also accounts for the time lag observed between the T flow and the increase in VPD. Similar observations were reported for Japanese plum [11]. Later in the day, the rate of transpiration of the fruit increases. The increased rate of transpiration and–possibly—the fruit osmotic potential act in the opposite direction, pulling apoplastic water into the fruit [34]. The xylem backflow thus reverts to an inflow from the tree into the fruit.

These findings demonstrate that xylem backflow can indeed occur in the pedicel of a European plum during the early hours after sunrise. However, this backflow is essentially limited to stage II of fruit development and occurs only occasionally during stage III, the stage when neck shrivel appears in the field. Furthermore, the net movement of water into the fruit (i.e. X+P+T) over any 24-hour period, was always positive. If xylem backflow were to be a factor in neck shrivel in European plum, the xylem backflow in that 24-hour period would have to exceed the X+P inflows. This never was the case. We suggest this makes xylem backflow a highly unlikely contributor to neck shrivel in European plum. Research should focus on other potential causal factors.

## Supporting information

S1 Fig Volume change of European plum during the 6 day experimental period. A, D, G: Un-treated fruit. B, E, H: Girdled fruit. C, F, I: Detached fruit. A-C: 76 days after full bloom (DAFB), D-F: 104 DAFB, G-I: 132 DAFB. Vertical dashed line indicates the time of treatment. In the morning of the fourth day, fruit pedicels either remained un-treated (A, D, G), or were steam-girdled (B, E, H) or detached (C, F, I), but all fruit remained *in situ* in the canopy. Data represent individual replicates.
(TIF)

S2 Fig Daily xylem (X), phloem (P) and transpiration (T) flows during a 3 day experimental period after steam-girdling or detachment. Representative diurnal time courses of xylem (X), phloem (P) and transpiration (T) flow rates in developing European plum fruit (A: stage II, 76, B: early stage III, 104, C: late stage III, 132 days after full bloom). Flows were calculated from net flows determined for un-treated control fruit, steam-girdled fruit and detached fruit.
(TIF)

S1 Data (ZIP)
