# Supplementary figures and images for: Xylem, phloem and transpiration flows in developing European plums
Source: PLoS One. 2021 May 20;16(5):e0252085. doi: 10.1371/journal.pone.0252085 (PMC8136697; doi:10.1371/journal.pone.0252085)

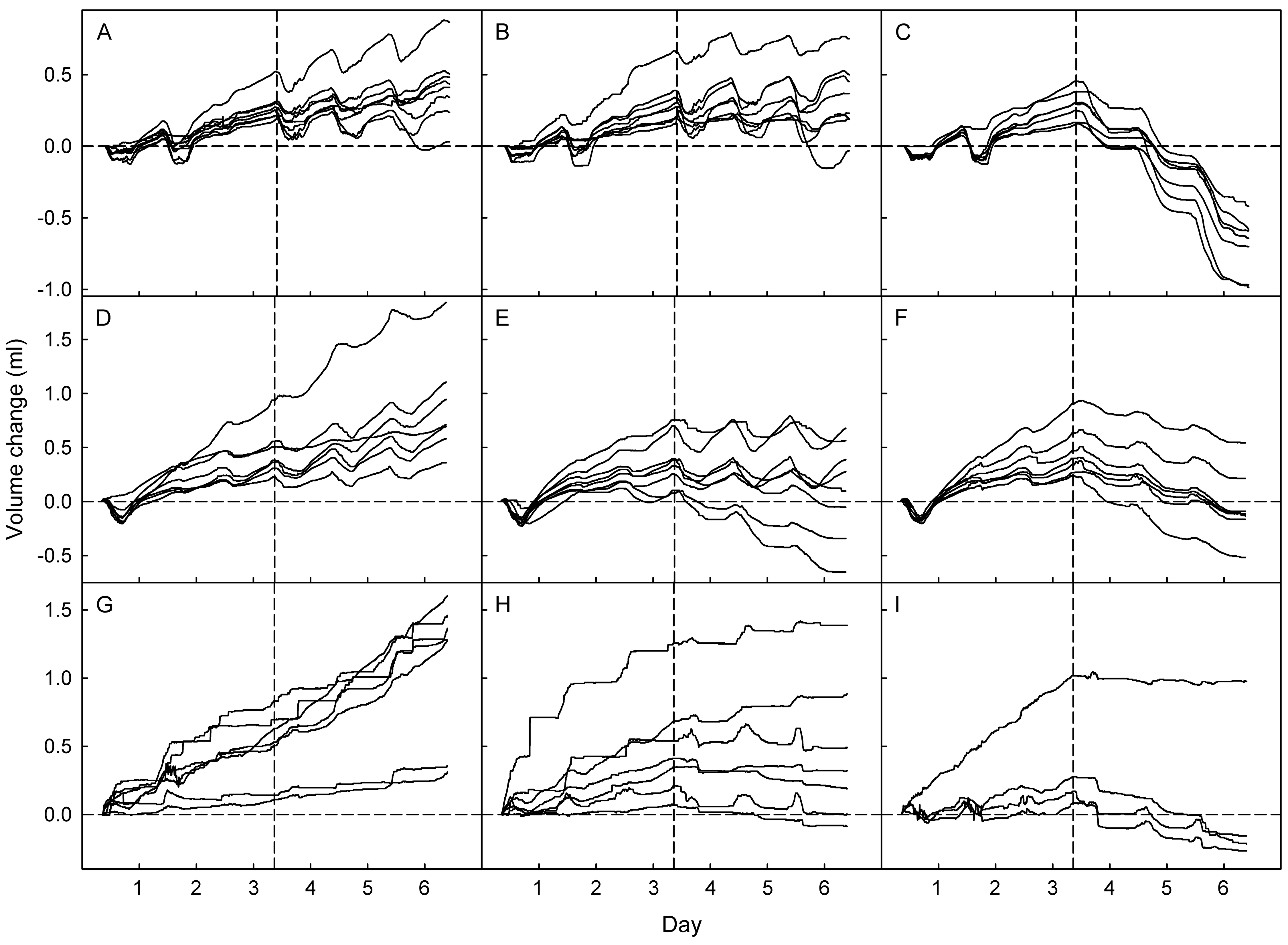

Supplement: S1 Fig — A, D, G: Un-treated fruit. B, E, H: Girdled fruit. C, F, I: Detached fruit. A-C: 76 days after full bloom (DAFB), D-F: 104 DAFB, G-I: 132 DAFB. Vertical dashed line indicates the time of treatment. In the morning of the fourth day, fruit pedicels either remained un-treated (A, D, G), or were steam-girdled (B, E, H) or detached (C, F, I), but all fruit remained in situ in the canopy. Data represent individual replicates. (TIF) [file pone.0252085.s001.tif]

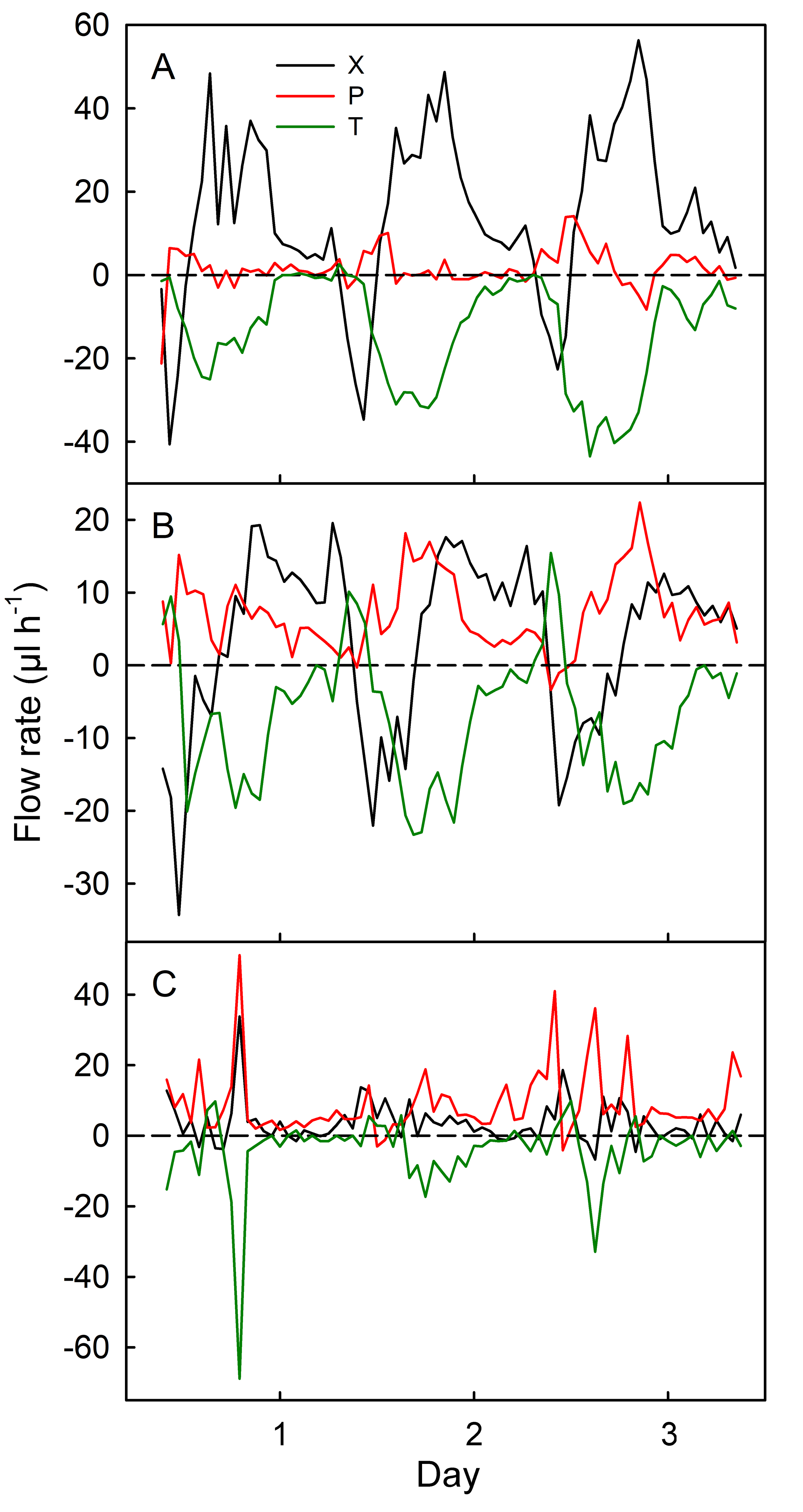

Supplement: S2 Fig — Representative diurnal time courses of xylem (X), phloem (P) and transpiration (T) flow rates in developing European plum fruit (A: stage II, 76, B: early stage III, 104, C: late stage III, 132 days after full bloom). Flows were calculated from net flows determined for un-treated control fruit, steam-girdled fruit and detached fruit. (TIF) [file pone.0252085.s002.tif]
